# How Statins May Protect against Alzheimer Disease

**DOI:** 10.1371/journal.pmed.0020022

**Published:** 2005-01-11

Epidemiological studies suggest that statins, a class of cholesterol-lowering drugs, may lower the risk for Alzheimer disease. The mechanism for this effect is unclear. Alzheimer disease is characterized by accumulation of amyloid deposits in the brain. These deposits are composed of amyloid-beta (Aβ) peptide, a protein fragment that is cleaved off from the amyloid precursor protein APP. APP can be cleaved in two different ways. Amyloidogenic (“amyloid generating”) cleavage by an enzyme called beta-secretase yields “sticky” Aβ peptides that aggregate to form deposits, whereas non-amyloidogenic cleavage by alpha-secretases generates soluble peptides that do not form deposits. Studies in animal models and cell culture suggest that statins might modulate APP processing and shift the balance toward “healthy” (non-amyloidogenic) cleavage.[Fig pmed-0020022-g001]

**Figure pmed-0020022-g001:**
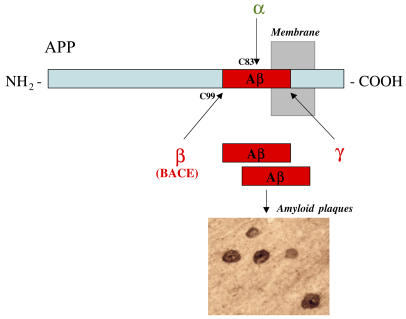
APP secretases (α, β, and γ)

In their quest to understand how statins affect APP processing, Sam Gandy and colleagues focused on a molecule called ROCK1, a kinase enzyme that had recently been implicated in APP processing. The theoretical link between statins and ROCK1 goes as follows: statins inhibit the isoprenoid pathway, isoprenoids are regulators of the enzyme Rho, and Rho in turn activates ROCK1. And while such potential connections could be drawn for any number of molecules, Gandy and colleagues went on to test whether statins exert their effect on APP cleavage by interfering with the isoprenoid/Rho/ROCK1 pathway.

Working in mouse neuroblastoma cells, they confirmed that two different statins increased healthy cleavage of APP. When they directly interfered with the isoprenoid/Rho/ROCK1 pathway by adding a drug that inhibits Rho activation, they saw effects similar to those of the statins (i.e., an increase in healthy cleavage). The same effects were seen when they transfected the cells with a dominant negative form of ROCK1 (which inactivates the normal ROCK1 molecules in the cell); this outcome shows that the pathway can influence APP cleavage. Most conclusively, when they added a version of ROCK1 that was constitutively (always) active, they reduced basal levels and abolished statin-stimulated levels of healthy cleavage.

Taken together, these results suggest that statins influence APP processing, at least in part, by modulating the isoprenoid pathway and inactivating the ROCK1 kinase. Future studies are necessary to determine whether this mechanism is actually responsible for the apparent clinical benefits of statins. Another question worth exploring further is whether ROCK1 might be a suitable target for therapeutic interventions that aim to decrease harmful, and promote healthy, cleavage of APP.

