# Correction: How Statins May Protect against Alzheimer Disease

**DOI:** 10.1371/annotation/be77eb35-1de9-4b45-b5be-fdf2e67a4fc7

**Published:** 2012-03-05

**Authors:** How Statins May Protect against Alzheimer Disease

An image that was published alongside another PLoS Medicine article (doi/10.1371/journal.pmed.0020024) and included in the shared PDF download here has been removed from the published PDF because of that image's copyright status. 

